# *Anaplasma phagocytophilum* and *Anaplasma marginale* Elicit Different Gene Expression Responses in Cultured Tick Cells

**DOI:** 10.1155/2009/705034

**Published:** 2009-07-15

**Authors:** Zorica Zivkovic, Edmour F. Blouin, Raúl Manzano-Roman, Consuelo Almazán, Victoria Naranjo, Robert F. Massung, Frans Jongejan, Katherine M. Kocan, José de la Fuente

**Affiliations:** ^1^Utrecht Centre for Tick-Borne Diseases (UCTD), Department of Infectious Diseases and Immunology, Faculty of Veterinary Medicine, Utrecht University, Yalelaan 1, 3584 CL Utrecht, The Netherlands; ^2^Department of Veterinary Pathobiology, Center for Veterinary Health Sciences, Oklahoma State University, Stillwater, OK 74078, USA; ^3^Facultad de Medicina Veterinaria y Zootecnia, Universidad Autónoma de Tamaulipas, Km. 5 carretera Victoria-Mante, CP 87000 Cd. Victoria, Tamaulipas, Mexico; ^4^Instituto de Investigación en Recursos Cinegéticos IREC (CSIC-UCLM-JCCM), Ronda de Toledo s/n, 13005 Ciudad Real, Spain; ^5^Division of Viral and Rickettsial Diseases, Centers for Disease Control and Prevention, Atlanta, GA 30333, USA; ^6^Department of Veterinary Tropical Diseases, Faculty of Veterinary Science, University of Pretoria, Private Bag X04, Onderstepoort 0110, South Africa

## Abstract

The genus *Anaplasma* (Rickettsiales: Anaplasmataceae) includes obligate tick-transmitted intracellular organisms, *Anaplasma phagocytophilum* and *Anaplasma marginale* that multiply in both vertebrate and tick host cells. Recently, we showed that *A. marginale* affects the expression of tick genes that are involved in tick survival and pathogen infection and multiplication. However, the gene expression profile in *A. phagocytophilum*-infected tick cells is currently poorly characterized. The objectives of this study were to characterize tick gene expression profile in *Ixodes scapularis* ticks and cultured ISE6 cells in response to infection with *A. phagocypthilum* and to compare tick gene expression responses in *A. phagocytophilum*- and *A. marginale*-infected tick cells by microarray and real-time RT-PCR analyses. The results of these studies demonstrated modulation of tick gene expression by *A. phagocytophilum* and provided evidence of different gene expression responses in tick cells infected with *A. phagocytophilum* and *A. marginale*. These differences in *Anaplasma*-tick interactions may reflect differences in pathogen life cycle in the tick cells.

## 1. Introduction

Ticks transmit pathogens that greatly impact both human and animal health [1]. The genus *Anaplasma* (Rickettsiales: Anaplasmataceae) includes obligate tick-transmitted intracellular organisms found exclusively within membrane-bound inclusions or vacuoles in the cytoplasm of both vertebrate and tick host cells [2, 3]. *A. marginale* infects cattle and wild ruminants and causes bovine anaplasmosis [2]. *A. phagocytophilum* infects humans and wild and domesticated animals [2, 4, 5] and is the causative agent of human, equine and canine granulocytic anaplasmosis and tick-borne fever of ruminants [5, 6]. In the United States, *A. phagocytophilum* is transmitted by *Ixodes scapularis* and *I. pacificus* [2, 4].

The ticks and the pathogens they transmit have evolved molecular interactions that affect their survival and life cycle [3]. The *A. phagocytophilum* outer membrane proteins that are involved in interactions with tick cells have been identified and partially characterized [7, 8]. Recently, we identified and characterized tick molecules that are involved in *A. marginale*-tick interactions, demonstrating that *A. marginale* affects the expression of tick genes essential for tick survival and pathogen infection and multiplication [9]. However, tick molecules that are affected by and participate in *A. phagocytophilum* infection and multiplication are currently poorly characterized [10, 11].

The objectives of this study were the characterization of tick gene expression profiles in *I. scapularis* ticks and cultured tick cells in response to infection with *A. phagocytophilum* and to compare tick gene expression responses in *A. phagocytophilum*- and *A. marginale*-infected cultured tick cells by microarray and real-time RT-PCR analyses. The results reported herein demonstrated modulation of tick gene expression by *A. phagocytophilum* and identified differentially expressed genes that are relevant for the understanding of basic biological questions of *A. phagocytophilum* life cycle in *I. scapularis*. The results also provided evidence of differences in tick gene expression in response to infection with *A. phagocytophilum* or *A. marginale*.

## 2. Materials and Methods

### 2.1. Uninfected and Anaplasma-Infected Ticks and Tick Cells

The *I. scapularis* nymphs uninfected and infected with *A. phagocytophilum* (Gaillard and Dawson strains) were obtained from a laboratory colony reared at the Centers for Disease Control and Prevention, Atlanta, Ga, USA. Tick larvae were fed on uninfected or infected mice, collected after feeding, and allowed to molt to nymphs. Animals were housed with the approval and supervision of the Institutional Animal Care and Use Committee.

The tick cell line ISE6, derived from *I. scapularis* embryos (provided by U.G. Munderloh, University of Minnesota, USA), was cultured in L15B medium as described previously for IDE8 cells [12], but the osmotic pressure was lowered by the addition of one-fourth sterile water by volume after Munderloh et al. [13]. The ISE6 cells were first inoculated with *A. phagocytophilum*- (NY18 isolate) infected HL-60 cells and maintained according to Munderloh et al. [13] until infection was established and routinely passaged. Infected ISE6 cells were frozen in liquid nitrogen and served as inoculum for uninfected cells. The ISE6 cells were initially infected with *A. marginale* (Virginia isolate) from a frozen inoculum of infected bovine erythrocytes. Following infection and routine passage, infected ISE6 cells were frozen in liquid nitrogen and served to infect uninfected ISE6 cells. For the current study each inoculum of infected cells was thawed and centrifuged, and the pellet was resuspended in culture medium and put on the ISE6 cells. When the infection reached approximately 80% of the tick cells, the monolayer was passaged onto uninfected ISE6 monolayers and maintained in L15B medium as described above. Monolayers of infected ISE6 cells were collected at different time points as described above. Uninfected cells were cultured in the same way but adding 1 mL of culture medium instead of infected cells. Collected cells were centrifuged at 10 000 × g for 3 minutes, and cell pellets were frozen in liquid N until used for RNA extraction.

The infection of ticks and tick cells with *A. phagocytophilum* or *A. marginale* was corroborated by major surface protein 4 (*msp4*) PCR [14, 15].

### 2.2. Microarray Analysis

Infected tick ISE6 cells were sampled at 6 days postinfection (dpi) with approximately 70% infected cells (separate cell cultures grown under similar conditions had >90% cells infected at 8 dpi). Uninfected cells were sampled at the same time point as infected cells to account for culture time effects. Total RNA was extracted from three *A. marginale*-infected, three *A. phagocytophilum*-infected, and three uninfected ISE6 cell cultures using the RNeasy Mini Kit (Qiagen) including the on-column DNA digestion with the RNase-free DNase set following manufacturer's instructions. RNA quality was checked by gel electrophoresis to verify the integrity of RNA preparations. Total RNAs (5 *μ*g) were labeled using the 3DNA Array900 kit with Alexa Fluor dyes (Genisphere, Hatfield, Pa, USA), Superscript II (Invitrogen, Carlsbad, Calif, USA), the supplied formamide-based hybridization buffer and 24 × 60 mm LifterSlips (Erie Scientific, Portmouth, NH, USA) according to the manufacturer's (Genisphere) instructions. The microarray was constructed with 768 random *I. scapularis* sequences enriched for genes differentially expressed after subolesin knockdown as previously described [16] (NCBI Gene Expression Omnibus (GEO) platform accession number GPL6394 and series number GSE10222). Eight pools of 12 clones each from an unsubtracted *I. scapularis* cDNA library and subolesin cDNA were also arrayed and used to validate normalization. Hybridization signals were measured using a ScanArray Express (PerkinElmer, Boston, Mass, USA), and the images were processed using GenePix Pro version 4.0 (Axon, Union City, Calif, USA). Ratios were calculated as *Anaplasma*-infected cells versus uninfected control cells. Preprocessing of data was accomplished using R-project statistical environment (http://www.r-project.org) and Bioconductor (http://www.bioconductor.org) and the LIMMA package as previously described [17]. This included (1) removal of data points where signal was less than the background plus two standard deviations in both channels, (2) removal of data points where signal was less than 200 RFU in both channels, (3) removal of poor quality spots flagged during image processing, (4) removal of spots with less than 50% valid biological and technical replicates, (5) log transformation of the background subtracted mean signal ratios, and (6) normalization using global Lowess intensity-dependent normalization. Normalized ratio values obtained for each probe were averaged across 3 biological replicates, and four technical replicates and significant differences were defined as *P*-value ≤ .05 and displaying an expression fold change greater than 2-fold in either *A. phagocytophilum* or *A. marginale* infected cells.

### 2.3. Sequence Analysis and Database Search

Partial sequences were determined for cDNA sequences identified as differentially expressed in the microarray analysis. Multiple sequence alignment was performed using the program AlignX (Vector NTI Suite V 8.0, InforMax, Invitrogen, Carisbad, Calif, USA) to exclude vector sequences and to identify redundant (not unique) sequences. Searches for sequence similarity were performed with the BLASTX program (http://www.ncbi.nlm.nih.gov/BLAST) against the nonredundant sequence database (nr) and databases of tick specific sequences (http://www.vectorbase.org/index.php; http://compbio.dfci.harvard.edu/tgi/).

### 2.4. Real-Time Reverse Transcription (RT)-PCR Analysis

Total RNA was extracted from uninfected and *A. phagocytophilum*-infected and *A. marginale*-infected ISE6 cells (three cultures each) and *A. phagocytophilum*-infected *I. scapularis* whole unfed nymphs (three groups of uninfected ticks, three groups of ticks infected with the Gaillard strain, and three groups of ticks infected with the Dawson strain with 10 nymphs each) using the RNeasy Mini Kit (Qiagen) including the on-column DNA digestion with the RNase-free DNase set following manufacturer's instructions. Infected tick cells were sampled at a single time point at 3 dpi with approximately 40% infected cells (companion cultures were terminal at 8 dpi) or at 2, 5, and 8 dpi. When included in the analysis, uninfected cells were sampled at the same time point as infected cells to account for culture time effects. Two primers were synthesized based on the sequences determined for candidate differentially expressed genes for real-time RT-PCR analysis. Real-time RT-PCR was done using the QuantiTec SYBR Green RT-PCR kit (Qiagen, Valencia, Calif, USA) and a Bio-Rad iQ5 thermal cycler (Hercules, Calif, USA) following manufacturer's recommendations. Reactions were done for 40 cycles and 30 seconds annealing using oligonucleotide primers and annealing temperatures described in Table 1. Negative controls included reactions without RNA. mRNA levels were normalized against tick *β*-actin or 16S rRNA using the comparative Ct method and compared between infected and uninfected tick cells and ticks or between *A. phagocytophilum*- and *A. marginale*-infected tick cells by Student's *t*-test (*P* = .05).

The *A. phagocytophilum* and *A. marginale* infection levels were evaluated in tick ISE6 cells at 2, 5, and 8 dpi by real-time PCR of *msp4* and normalizing against tick 16S rDNA sequences using the QuantiTec SYBR Green PCR kit (Qiagen) in an iQ5 thermal cycler (Bio-Rad) as described above. Known amounts of the full length *A. phagocytophilum* and *A. marginale msp4* PCR product were used to construct a standard for quantitation of pathogens per cell.

### 2.5. Nucleotide Sequence Accession Numbers

The nucleotide sequences of the ESTs reported in this paper have been deposited in the GenBank database under accession numbers FL685631-FL685658.

## 3. Results

### 3.1. A. phagocytophilum Modulates Gene Expression in Infected I. scapularis Nymphs and Tick ISE6 Cells

The infection with *A. marginale* has been shown to modulate tick gene expression [9]. However, the effect of *A. phagocytophilum* infection on tick gene expression is unknown. Here, two experimental approaches were used to characterize gene expression profiles in tick cells infected with *A. phagocytophilum*. In the first approach, tick gene expression was characterized by microarray analysis of RNA from infected and uninfected tick ISE6 cells. In the second approach, genes identified as differentially expressed in tick IDE8 cells and ticks infected with *A. marginale* were used to characterize the effect of *A. phagocytophilum* on tick gene expression in infected *I. scapularis* nymphs and tick ISE6 cells by real-time RT-PCR.

The microarray analysis showed in *A. phagocytophilum*-infected tick ISE6 cells the upregulation of genes C4B10 with homology to von Willebrand factor and R1E12 with homology to ribosomal protein L32, C4A10, C3C3, C4A1, and C3D9 with unknown function and the downregulation of genes C3B2 with homology to an aspartic protease and R2A12, C3A7, R2G1, R2D6, C3C11, and R3D4 with unknown function (Table 2). The expression of other genes with homology to troponin I (C2E6), putative secreted salivary protein (C4G3), and ML domain-containing protein (R4G5) did not change after infection of tick ISE6 cells with *A. phagocytophilum* (Table 2).

**Figure 1:**
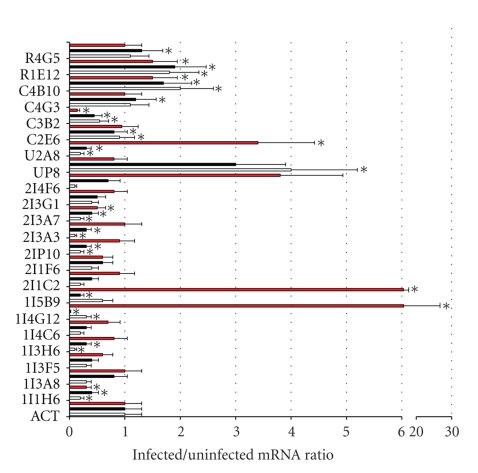
Differential gene expression in *A. phagocytophilum*-infected *I. scapularis* ticks and cultured tick ISE6 cells. Real-time RT-PCR was done on uninfected and infected *I. scapularis* nymphs (three groups each of uninfected ticks, infected ticks (Gaillard strain; black bars) and infected ticks (Dawson strain; white bars) with 10 nymphs each) and uninfected and NY18 isolate-infected tick ISE6 cells (three independent cultures each; red bars). Bars represent the ratio between infected normalized Ct values/uninfected average normalized Ct values (+SD). The mRNA levels were normalized against tick *β*-actin (ACT) and compared between infected and uninfected ticks and tick cells by Student's *t*-test (*P ≤ .05).

The mRNA levels of selected genes differentially expressed in *A. phagocytophilum*-infected tick ISE6 cells were evaluated by real-time RT-PCR in infected and uninfected cells (Figure 1). Similar to microarray hybridization results, the analysis of mRNA levels by real-time RT-PCR showed significant upregulation of C4B10 (von Willebrand factor) and R1E12 (ribosomal protein L32) and downregulation of C3B2 (aspartic protease) in tick ISE6 cells infected with *A. phagocytophilum* (Figure 1). The mRNA levels of genes identified previously as differentially expressed in tick IDE8 cells and ticks infected with *A. marginale* [9] were also evaluated by real-time RT-PCR in infected and uninfected tick ISE6 cells. The mRNA levels of genes differentially expressed in *A. marginale*-infected ISE6 cells were similar to those reported previously in infected IDE8 cells [9] and data not shown. The results in *A. phagocytophilum*-infected ISE6 cells showed that pathogen infection significantly upregulated the expression of U2A8 (signal sequence receptor delta), 1I5B9 (ixodegrin-2A RGD containing protein), and 1I4G12 (unknown function) and downregulated the expression of 2I3A7 (NADH-ubiquinoe oxidoreductase) and 1I1H6 (glutathione S-transferase (GST)) in tick ISE6 cells (Figure 1). 

The expression of selected genes was also analyzed in *I. scapularis* nymphs infected with two different *A. phagocytophilum* strains (Figure 1). In *I. scapularis* nymphs, the expression of C4G3 (putative secreted salivary protein), C4B10 (von Willebrand factor), R1E12 (ribosomal protein L32), and R4G5 (ML domain-containing protein) was significantly upregulated, and the expression of U2A8 (signal sequence receptor delta), UP8 (ferritin), 2I3A7 (NADH-ubiquinoe oxidoreductase), 2I3A3 (gamma actin-like protein), 2IP10 (ubiquitin C variant 5-like), 1I5B9 (ixodegrin-2A RGD containing protein), 1I1H6 (GST), C2E6 (troponin I), C3B2 (aspartic protease), and 1I4G12 and 1I3H6 with unknown function was significantly downregulated. Interestingly, the mRNA levels were similar in *I. scapularis* nymphs infected with two different strains of *A. phagocytophilum* but differed from those obtained in infected ISE6 cells for some genes such as U2A8, 1I5B9, 1I4G12, C2E6, C4G3 and R4G5 (Figure 1).

### 3.2. Differential Gene Expression in A. phagocytophilum-Infected I. scapularis Nymphs and ISE6 Cells Differed from That Observed after A. marginale Infection

**Figure 2:**
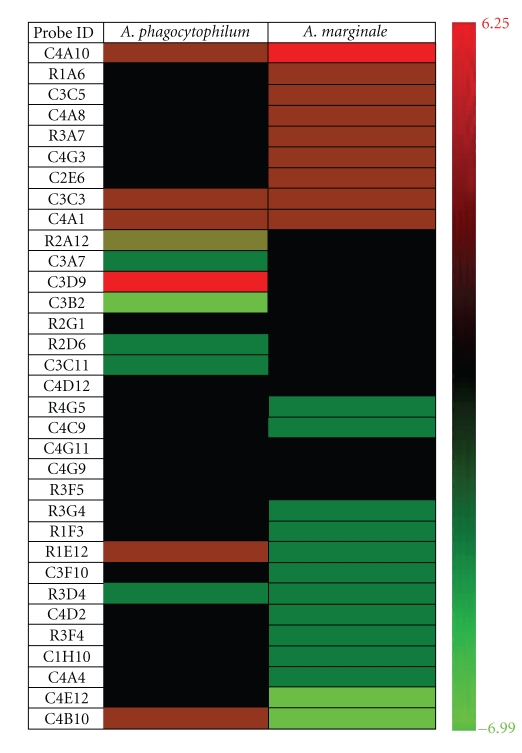
Effect of *A. phagocytophilum* and *A. marginale* infection on tick ISE6 cells gene expression. Total RNA was extracted from three *A. marginale*-infected, three *A. phagocytophilum*-infected, and three uninfected ISE6 cell cultures. The expression fold change was determined by microarray hybridization at 6 days postinfection (dpi) (approximately 70% infected cells; companion cultures were terminal at 8 dpi). Uninfected cells were sampled at the same time point as infected cells to account for culture time effects. Ratios were calculated as *Anaplasma*-infected cells versus uninfected control cells. Normalized ratio values obtained for each probe were averaged across 3 biological replicates and four technical replicates and only entries displaying a significant (*P* ≤ .05) expression fold change >2 in either *A. phagocytophilum*- or *A. marginale*-infected cells are shown. Clone ID (library plate and well) are shown. The graph was constructed with the HCE software (http://www.cs.umd.edu/hcil/hce/hce3.html).

**Figure 3:**
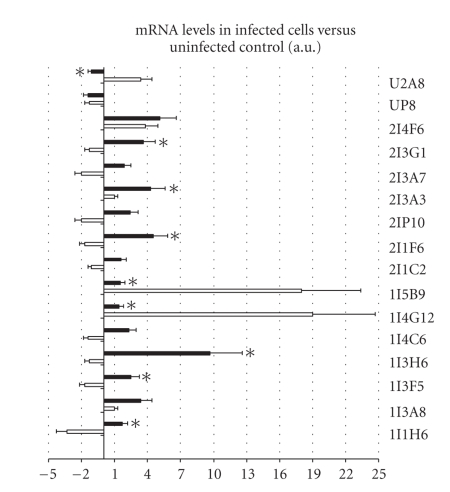
Effect of *A. phagocytophilum* and *A. marginale* infection on tick ISE6 cells gene expression. The mRNA levels were compared between *A. phagocytophilum*- (white bars) and *A. marginale*- (black bars) infected tick ISE6 cells by real-time RT-PCR. Bars represent the ratio between infected normalized Ct values and uninfected average normalized Ct values (+SD). The mRNA levels were normalized against tick 16S rRNA and compared between *A. phagocytophilum*- and *A. marginale*-infected tick cells by Student's *t*-test (*P ≤ .05). Positive and negative values denote upregulation and downregulation, respectively, with respect to uninfected controls.

The results reported herein showed that gene expression profiles were different for *A. phagocytophilum*- and *A. marginale*-infected tick cells (Figures  2  and  3). The microarray analysis in infected and uninfected tick ISE6 cells showed that the expression of genes with homology to internal transcribed spacer 1 (probe C3C5), putative secreted salivary protein (C4G3), troponin I (C2E6), aspartic protease (C3B2), ML domain-containing protein (R4G5), H13-prov protein (C4C9), ribosomal protein L32 (R1E12), putative secreted protein (C3F10), ribosomal protein S17 (C4E12), von Willebrand factor (C4B10), and sequences with unknown function (R1A6, C4A8, R3A7, R2A12, C3A7, C3D9, R2D6, C3C11, R3G4, R1F3, C4D2, R3F4, C1H10, C4A4) was different between *A. phagocytophilum*- and *A. marginale*-infected cells (Figure 2). The expression of other genes changed in a similar way after infection of tick ISE6 cells with *A. marginale* or *A. phagocytophilum* (C4A10, C3C3, C4A1, R2G1, C4D12, C4G11, C4G9, R3F5, R3D4; Figure 2).

By real-time RT-PCR, the mRNA levels of U2A8 (signal sequence receptor delta), 2I3G1 (proteasome 26S subunit, non-ATPase), 2I3A3 (gamma actin-like protein), 2I1F6 (hematopoietic stem/progenitor cells protein-like), 1I5B9 (ixodegrin-2A RGD containing protein), 1I4G12 (unknown function), 1I3H6 (unknown function), 1I3F5 (ubiquitin), and 1I1H6 (GST) were significantly different between *A. phagocytophilum*- and *A. marginale*-infected ISE6 cells collected at 3 dpi with approximately 40% infected cells (Figure 3). Except for U2A8, 1I5B9, 1I4G12, C2E6, C4G3, and R4G5 which had different mRNA levels in *A. phagocytophilum*-infected nymphs and tick ISE6 cells (Figure 1), the mRNA levels of the studied genes were also different between *A. phagocytophilum*-infected *I. scapularis* nymphs and *A. marginale*-infected tick cells (data not shown).

**Figure 4:**
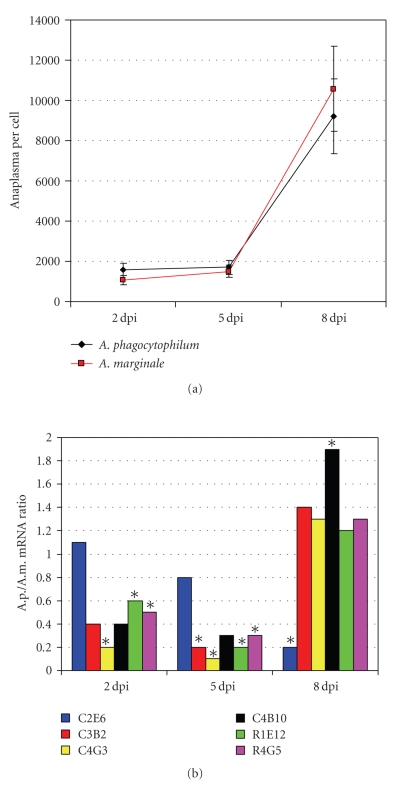
Comparison between differential gene expression in *A. phagocytophilum*- and *A. marginale*-infected tick ISE6 cells at different time points after infection. Studies were done on *A. phagocytophilum*- (A.p.-) and *A. marginale*- (A.m.-) infected tick ISE6 cells (two independent cultures each) at 2, 5, and 8 days postinfection (dpi) with approximately 30%–40%, 60%–70%, and >90% infected cells, respectively. (a) The A.p. and A.m. infection levels were evaluated by real-time PCR of *msp4* and normalized against tick 16S rDNA. Known amounts of the full length A.p. and A.m. *msp4* PCR product were used to construct a standard curve for quantitation of pathogens per cell. Data represent average ± SD. (b) The mRNA levels of selected genes were evaluated by real-time RT-PCR and normalized against tick 16S rRNA. Bars represent the ratio between average Ct values in A.p.-infected cells/average Ct values in A.m.-infected cells. The mRNA levels were compared between A.p.- and A.m.-infected tick cells by Student's *t*-test (*P ≤ .05). Identical mRNA levels in *A. phagocytophilum* and *A. marginale* infected cells equal one.

Because the kinetics of differentially expressed genes may vary with *Anaplasma* infection levels, the expression of selected genes was compared by real-time RT-PCR in *A. phagocytophilum*- and *A. marginale*-infected ISE6 cells collected at 2, 5, and 8 dpi (Figure 4(a)). The results showed time-dependent variation in the mRNA ratios of studied genes between *A. phagocytophilum*- and *A. marginale*-infected cells (Figure 4(b)). However, significant differences were observed between *A. phagocytophilum* and *A. marginale* infection at all time points, thus suggesting that differences in gene expression profiles elicited by these pathogens are present throughout the infection cycle in tick ISE6 cells.

## 4. Discussion

We have shown previously that *A. marginale* modulates gene expression in infected ticks and tick cells [9]. In the experiment reported herein we hypothesized that *A. marginale* and *A. phagocytophilum* may elicit similar gene expression responses in infected cultured tick cells. To test this hypothesis, gene expression profiles were compared between *A. phagocytophilum* and *A. marginale* in infected tick ISE6 cells. The results showed that *A. phagocytophilum* modulates gene expression in infected *I. scapularis* nymphs and cultured tick ISE6 cells but with different gene expression profiles when compared with *A. marginale*. These results suggested that *A. marginale* and *A. phagocytophilum* produce different differential gene expression profiles in infected tick cells. These differences in *Anaplasma*-tick interactions may reflect differences in pathogen developmental cycle in the tick cells. Alternatively, differences in gene expression profiles between *A. phagocytophilum*- and *A. marginale*-infected tick ISE6 cells may be due to the fact that *I. scapularis* is not a natural vector of *A. marginale*. However, *I. scapularis*-cultured cells have shown to provide functionally relevant data for the study of tick-*A. marginale* interactions [9]. The differences in gene expression between *A. marginale*- and *A. phagocytophilum*-infected tick cells could be attributed to nonspecific responses to the presence of bacterial components that differ between the two *Anaplasma* species While this explanation is potentially possible, it is more likely that gene expression profiles resulted from *Anaplasma* intracellular infection because at least for some genes differential expression persisted until 8 dpi, when >90% cells were infected. Taken together the results reported here consistently provided differences in gene expression profiles between *A. phagocytophilum*- and *A. marginale*-infected tick cells. Importantly, sampling time points during *Anaplasma* infection of tick ISE6 cells may be important to characterize the expression of particular genes. Although not addressed in this study, these differences may be also present during tick feeding and development.

The genes differentially expressed in *I. scapularis* nymphs and tick ISE6 cells infected with *A. phagocytophilum* included some genes such as GST and ferritin shown previously to affect *A. marginale* infection and/or multiplication in ticks and/or tick cells [9]. However, while GST and ferritin were upregulated and downregulated after *A. marginale* infection, respectively, they were regulated in the opposite direction in *A. phagocytophilum*-infected ticks and tick cells. GST, ferritin, and aspartic protease (C3B2), also found to be differentially expressed in *A. phagocytophilum*-infected ISE6 cells, have been reported to be regulated by tick feeding or infection with other pathogens [12–18]. Other genes differentially expressed after *A. phagocytophilum* infection such as U2A8 (signal sequence receptor delta), 1I5B9 (ixodegrin-2A RGD containing protein), 2I3A7 (NADH-ubiquinone oxidoreductase), 2IP10 (ubiquitin C variant 5-like), 2I3A3 (gamma actin-like protein), C4B10 (von Willebrand factor), C2E6 (troponin I), and R1E12 (ribosomal protein L32) constitute new findings and may be involved in infection and/or multiplication of the pathogen in ticks or are part of tick cell immune response to moderate infection levels. As shown previously for *A. marginale* [9], RNA interference experiments may help to characterize the function of differentially expressed genes during *A. phagocytophilum* infection of ticks and tick ISE6 cells.

The expression of selected genes was analyzed in *I. scapularis* ISE6 cells and nymphs infected with *A. phagocytophilum*. These experiments allowed us to compare the results of gene expression in vitro and in vivo. The nymphs were selected for analysis because this stage plays an important role during pathogen transmission to humans [5]. The ISE6 cell line was obtained from embryos of *I. scapularis*, one of the natural vectors of *A. phagocytophilum* [19]. However, this cell line is heterogeneous in the cell types represented [19], which may have different susceptibility and response to pathogen infection. Although cultured tick cells have been shown to be a good model for the study of tick-*Anaplasma* interactions [19–21], these results demonstrated that differences may exist between *I. scapularis*-cultured tick cells and nymphs in the mRNA levels of certain genes, at least under the experimental conditions used herein. These differences may account for differences in gene expression between infected ISE6 cells and tick whole nymph tissues and/or due to differences in the infection levels in both systems. An additional source of potential differences in gene expression between *I. scapularis* ISE6 cells and nymph tissues could be attributed to the fact that different cells types may be infected in both systems resulting in different responses to infection. Finally, although less likely, these differences may be related to differences between the NY18 isolate used to infect ISE6 cells and the Gaillard and Dawson strains used to infect *I. scapularis* nymphs.

Recent studies have characterized *A. marginale* and *A. phagocytophilum* proteins that are involved in interactions with tick cells [7, 8, 22]. However, tick-*Anaplasma* coevolution also involves genetic traits of the vector as demonstrated recently in studies on the role of tick proteins in the infection and transmission of *A. marginale* [9, 11] and *A. phagocytophilum* [10, 11]. Furthermore, genetic factors have been associated with intraspecific variation in vector competence for a variety of vector-borne pathogens, including *A. phagocytophilum* [23] and *A. marginale* [24, 25].

## 5. Conclusions

In summary, we have characterized the gene expression profile in *A. phagocytophilum*-infected *I. scapularis* nymphs and cultured ISE6 cells. Interestingly, differential gene expression seems to differ between *A. marginale* and *A. phagocytophilum* infected cultured tick cells. Future experiments would provide detailed information on the role of these genes during *A. phagocytophilum* life cycle in ticks. These results provide fundamental information toward understanding tick-*Anaplasma* interactions and may lead to formulations of new interventions for the prevention of the transmission of tick-borne pathogens.

##  OMB Disclaimer

The findings and conclusions in this report are those of the authors and do not necessarily represent the views of the CDC or the United States Department of Health and Human Services.

## Figures and Tables

**Table 1:** RT-PCR oligonucleotide primers and conditions for the characterization of the expression profiles of differentially expressed tick genes.

Gene ID^(a)^	Upstream/downstream primer sequences	PCR annealing temperature
1I1H6	GGTACATGGAATCCGACTGC	54°C
	GTCCCCTTTTGCTTCGACTT	
1I3A8	GACGCAAAACTTCCTTCGAG	54°C
	GCACTTTCCAAGAGCCTGAC	
1I3F5	GCTTTCACGTTTTCGATGGT	50°C
	GGCAAAGATCCAAGACAAGG	
1I3H6	GCCTAGGGAGGACGTCGTAG	50°C
	ACGTGGAACACATCGAGTCA	
1I4C6	AATGCGAGACACTGGAGGAC	50°C
	AATCCAGGAATGTTGCCAAG	
1I4G12	GACGGACCTTGTCCGACTAC	53°C
	ATTCCCTCCTTGTCCTGGAT	
1I5B9	CGTCCCCTTCTGTGGAATTA	53°C
	TCATCGTTGTTCTGGTCTCG	
2I1C2	GAGACCATCAAGTGGCTGGA	53°C
	CTTGGTGATGATGGGGTTG	
2I1F6	CAACCCCAAGATCGTCAACT	53°C
	ACGCGTCCTTACGTTTCACT	
2IP10	TCTTGCCGGTCAGAGTCTTT	53°C
	GAAGGCGAAAATTCAGGACA	
2I3A3	TAAAACCCCTTTCCCCACTT	53°C
	GCACTCGAACCTAGCAAACC	
2I3A7	TCGACTCTGTTCAGGAGGAAG	53°C
	GGTCCAAATGGCAGAGCAT	
2I3G1	AGGAAGTGCACGATGATGG	54°C
	GGTTGGTTATCCTCTGGGAGA	
2I4F6	CTTTCTTGCCGTGCTTCTTT	53°C
	GCTCAACTTCCTCGTCGTTC	
UP8	CCTCCCTCGCTAACCTCTCT	54°C
	ATCGTCACGGTCGAAGTAGC	
U2A8	GCTCATCGTCGCCAACAT	54°C
	GAGTTCCTCCGTCCAGCTC	
C2E6	GTAAAGCCCGCTCTCAAGAA	53°C
	CATTCGGGTTTGTCCACAG	
C3B2	GAGTAGTGCCCGTCTTCGAC	53°C
	AGGTGATGCTGCCCTTGTAG	
C4G3	AACTGCCTTGGAGTTGCAGT	53°C
	CTTGTGTCCCAGGTGGAAGT	
C4B10	GTTCTTCTAACGGCCACTGC	53°C
	AGTCTTTGGTGCAAGCGAGT	
R1E12	ATGTGAAGCTGAGGCCAAAC	53°C
	GGAATTCGATTAGCGTGGTC	
R4G5	CCTTCCCTGCAATGTCAAAT	53°C
	CACAAGTGGGCAATCAACAC	
Beta actin	GAGAAGATGACCCAGATCA	50°C
(AF426178)	GTTGCCGATGGTGATCACC	
16S rRNA	GACAAGAAGACCCTA	42°C
(L34293)	ATCCAACATCGAGGT	

^(a)^IDs for *I. scapularis* genes are described herein and in de la Fuente et al. [9].

**Table 2:** Microarray analysis of gene expression profile in *A*.* marginale*- and *A*.* phagocytophilum*-infected and -uninfected tick ISE6 cells.

Probe ID^(a)^	Description^(b)^	*A. marginale* infection versus control		*A. phagocytophilum* infection versus control	
		Fold change^(c)^	SD^(d)^	Fold change^(c)^	SD^(d)^

C4A10	No homolog found	6.249	0.000	2.208	0.294
R1A6	No homolog found	2.539	0.162	1.049	0.346
C3C5	[Genbank:L22271] internal transcribed spacer 1 (*Ixodes dammini*)	2.406	1.211	1.383	0.000
C4A8	No homolog found	2.239	0.165	1.188	0.442
R3A7	No homolog found	2.209	0.805	−1.384	0.562
C4G3	[Genbank:AAY66629] putative secreted salivary protein (*Ixodes scapularis*)	2.167	0.326	1.037	0.614
C2E6	[Genbank:ABB89211] troponin I protein (*Rhipicephalus haemaphysaloides*)	2.040	0.400	−1.068	0.442
C3C3	No homolog found	1.916	0.559	3.422	1.037
C4A1	No homolog found	1.857	0.000	2.121	0.517
R2A12	No homolog found	1.199	0.232	−2.219	0.450
C3A7	No homolog found	1.135	0.282	−3.028	0.141
C3D9	[Genbank:XP_791420] hypothetical protein (*Strongylocentrotus purpuratus*)	1.076	0.335	4.875	2.069
C3B2	[Genbank:BAE53722] aspartic protease (*Haemaphysalis longicornis*)	−1.311	0.330	−6.986	0.379
R2G1	No homolog found	−1.497	0.495	−2.086	0.826
R2D6	No homolog found	−1.538	0.309	−2.440	0.563
C3C11	No homolog found	−2.053	0.401	−2.477	0.488
C4D12	No homolog found	−2.066	0.547	−1.022	0.533
R4G5	[Genbank:AAP84098] ML domain-containing protein (*Ixodes ricinus*)	−2.066	0.161	1.020	0.266
C4C9	[Genbank:AAH56007] H13-prov protein (*Xenopus laevis*)	−2.070	0.270	1.081	0.689
C4G11	[Genbank:EAA09467] ENSANGP00000010016 (*Anopheles gambiae*)	−2.093	0.550	−1.193	0.598
C4G9	No homolog found	−2.095	0.402	−1.598	0.888
R3F5	[Genbank:AAY66764] putative secreted salivary protein (*Ixodes scapularis*)	−2.118	0.310	−1.037	0.320
R3G4	No homolog found	−2.292	0.259	1.090	0.415
R1F3	No homolog found	−2.339	0.570	−1.344	0.855
R1E12	[Genbank:NP_001119682] ribosomal protein L32 (*Acyrthosiphon pisum*)	−2.379	0.000	2.488	0.000
C3F10	[Genbank:AAM93633] putative secreted protein (*Ixodes scapularis*)	−2.386	0.545	−1.647	0.315
R3D4	No homolog found	−2.529	1.046	−2.377	0.518
C4D2	No homolog found	−2.702	0.860	1.043	0.972
R3F4	No homolog found	−2.928	0.298	1.174	0.396
C1H10	No homolog found	−3.341	0.307	−1.057	0.000
C4A4	No homolog found	−3.678	1.181	−1.430	0.331
C4E12	[Genbank:AAY66942] ribosomal protein S17 (*Ixodes scapularis*)	−3.964	0.822	1.430	0.993
C4B10	[Genbank:AAQ01562] von Willebrand factor (*Ixodes ricinus*)	−4.422	0.000	2.413	0.000

^(a)^Probe ID (library plate and well) identifies sample (clone) in stock plates. ^(b)^Description of the probe based on top (best) BLASTX alignment. ^(c)^Fold change is the fold change of Lowess intensity-dependent normalized log2 ratio of valid background-corrected means averaged between valid replicates. Only entries displaying an expression change greater than 2-fold and *P* < .05 in either *A. phagocytophilum* or *A. marginale* infected cells are shown. Positive and negative values correspond to genes upregulated and downregulated in infected cells, respectively. ^(d)^SD is the standard deviation determined from the normalized average log2 ratio but determined on data from valid spots only.
